# Padel vs. tennis doubles: a comparison of performance demands and game attributes

**DOI:** 10.3389/fspor.2025.1540424

**Published:** 2025-06-18

**Authors:** Rūtenis Paulauskas, Domantas Šakinis, Bruno Figueira

**Affiliations:** ^1^ Educational Research Institute, Education Academy, Vytautas Magnus University, Kaunas, Lithuania; ^2^ Departamento de Desporto e Saúde, Escola de Saúde e Desenvolvimento Humano, Universidade de Évora, Évora, Portugal; ^3^ Comprehensive Health Research Centre (CHRC), Universidade de Évora, Évora, Portugal

**Keywords:** external load, speed zones, groundstrokes, volleys, rallies

## Abstract

**Introduction:**

This study investigates differences in performance demands and match characteristics between padel and tennis doubles.

**Method:**

Eight national-level male players (age of 27.0 ± 7.4 years, height of 186.3 ± 7.7 cm, body mass of 81.5 ± 10.7 kg, training frequency of tennis 4.8 ± 1.6 and padel 4.9 ± 1.4 h/week) participated in a total of 12 simulated matches, consisting of six tennis doubles and six padel sessions. The sessions were analyzed to assess various performance and physiological metrics. Match analysis focused on rally duration, strokes per rally, and movement characteristics, measured through standardized methods. Statistical comparisons were conducted using linear mixed models to identify significant differences between performance demands and match characteristics that define Padel and Tennis players.

**Results:**

Results indicate that tennis involves greater movement distances, higher speeds, more sprints, and longer rest intervals between rallies. In contrast, padel matches featured a higher total number of rallies, more frequent volleys and ground strokes, and longer play durations. All variables compared between Tennis and Padel showed statistical differences (*p* < 0.05). Despite these disparities, average heart rate and lactic acid responses were comparable across both sports, indicating similar physiological demands.

**Discussion:**

These findings highlight the importance of sport-specific training regimens tailored to the unique requirements of each sport. Practical applications include optimizing training to enhance endurance and tactical adaptability for Padel players, while emphasizing explosive power and recovery strategies for Tennis athletes.

## 1 Introduction

Padel and tennis share several similarities as racquet sports, including a common scoring system, gameplay dynamics, a multidirectional playing style, and technical-tactical skills like volleys, lobs, and smashes, which are transferable to match situations (1). Both sports appeal to individuals of all genders and a broad age spectrum, making them ideal choices for those seeking performance, a blend of physical activity, and community engagement (2). However, distinct differences exist, shaped by variables including playing surfaces, court dimensions, net height, techniques, and equipment. Padel is widely regarded as less physically demanding than tennis due to its smaller court size, the use of walls to sustain rallies, and the slower speed of the ball speed (3). Tennis imposes unique physiological and psychological demands due to its combination of explosive movements, intermittent workloads, stroke-specific ball speeds and technical precision (4, 5). Both tennis and padel are characterized by intermittent high-intensity efforts interspersed with periods of active or passive recovery, relying heavily on a combination of aerobic and anaerobic metabolic systems. In tennis, short-duration, high-power actions such as serves, sprints, and directional changes are underpinned predominantly by phosphagen and glycolytic energy systems, particularly during intense rallies or in singles match play (6). However, between-point recovery allows for partial resynthesis of phosphocreatine and lactate clearance via aerobic mechanisms, making oxidative metabolism an essential contributor to repeated performance capacity and recovery (7). The energetic demands thus reflect a hybrid profile, with anaerobic bursts being superimposed on a largely aerobic base that supports prolonged match durations and recovery between high-intensity phases. In contrast, the energy profile of padel aligns more closely with the characteristics of small-sided intermittent sports. The use of walls to prolong rallies, reduced court size, and relatively shorter sprints result in a more continuous engagement at moderate intensities. Studies suggest that although padel entails frequent changes of direction and technical executions, it is largely sustained through oxidative pathways with occasional engagement of glycolytic energy, particularly during longer exchanges or repeated lobs and smashes (7). The sport's reduced external load compared to tennis doubles minimizes the contribution of alactic pathways and favors an aerobic-glycolytic energy interplay. Therefore, the training regimens for these sports must account for their distinct metabolic profiles by emphasizing explosive anaerobic capacity in tennis, and aerobic efficiency and technical precision under moderate fatigue in padel.

Internal load analysis suggests that padel players sustain heart rates within 70%–80% of their maximal capacity during play (8). Blood lactate (Bla) levels in padel are moderate, with an average of 2.6 ± 1.3 mmol/L measured at the end of matches, indicating a mix of aerobic and anaerobic energy contributions during play (3). Higher-level players report lower RPE scores (e.g., 3.2 ± 2.0) compared to lower-level players (5.1 ± 1.7), likely due to better technical and tactical efficiency (8). Furthermore, studies indicate that post-match neuromuscular changes occur, yet there is no significant decline in overall performance (9). Mental fatigue, particularly among elite male players, has been shown to impair padel-specific accuracy during both training and competitive play (10). Additionally, the distance covered per point correlates positively with the number of points played, reinforcing the connection between player activity levels and match duration (11). In parallel, the workload of tennis matches is influenced by several factors, including player skill, playing style, and court surface, which highlights the necessity for training regimens that replicate match-specific conditions (12). On average, only about 33% of match time is spent in high-intensity zone (above 85% of maximum HR), reflecting the reduced physical demands of doubles tennis due to shorter rallies, shared court coverage, and more strategic positioning (13). Bla levels are generally low in doubles tennis, averaging between 1 and 4 mmol/L, reflecting the sport's intermittent nature and adequate recovery time between points (12). A comparative analysis of clay vs. hard court matches reveals significant differences in distance covered, intensity, and accelerations, with clay courts requiring more high-intensity movements (4). Further investigation into physiological responses during serve and return games, as well as differences between winners and losers, found no substantial differences in physiological markers (14). Studies conducted with subjects of varying levels, under different conditions, and measuring different parameters have not provided a clear, objective distinction between the physiological demands of padel and tennis. Therefore, conducting a direct comparison of external and internal load measures could effectively fill this gap by providing a comprehensive understanding of the physical demands and match responses in both sports.

An analysis of game attributes revealed that padel matches typically have sets lasting about 30–40 min, with variations influenced by factors such as the level of play, gender, and match dynamics (15). The ball is in play for about 30%–35% of the total match duration, with longer rally times observed in women's matches compared to men's (15). Professional padel matches typically feature 10–11 strokes per rally for men, depending on the study and competition level (16). While recreational players generally perform 4–6 strokes per rally, reflecting shorter rallies and less technical gameplay (5). Doubles tennis matches tend to be shorter than singles due to quicker points and frequent net play, which reduces rally length (17). In doubles tennis, the duration of a set typically ranges from 30 min to 1 h, depending on factors such as the level of competition, rally length, and scoring format (13). In men's doubles, the average rally length is approximately 2.5 strokes per point, with more than 81.6% of points concluding in three shots or fewer, and only 1.1% extending to 10 shots or more (18). Padel and tennis share common match variables, but the complexity of previous studies, varying objectives, and differing conditions make it challenging to directly compare these sports without a detailed examination of their game-specific characteristics.

A couple of systematic studies have been conducted on activity profiles and physiological responses during match play in popular racquet sports, but none of the samples included tennis doubles and padel (1). To date, only one study has directly compared padel and tennis revealing that padel imposes a distinctive match load on players (19). This load differs significantly from singles tennis but closely resembles that of doubles tennis. Furthermore, cardiovascular and physical demands vary substantially across different game formats (19). Recent advancements in wearable inertial sensors are revolutionizing kinematic analysis in sports, offering athletes real-time feedback to enhance performance efficiency (20). In parallel, the integration of Local Positioning System (LPS) technology and specialized training methodologies has shown substantial benefits for improving multiple aspects of tennis and padel performance.

Despite these advancements, research into skill transfer between tennis and padel, as well as player adaptation to environmental and task constraints, remains limited. Transitioning between these sports is shaped by a complex interaction among the individual, their environment, and the tasks at hand (21). This process often involves modifications to movement patterns, physical and physiological demands, perceptual and conceptual skills, and mental or cognitive capacities (22). A comprehensive understanding of the physical demands and game-specific characteristics of both sports is essential for optimizing player performance, refining training protocols, and identifying transferable skills that can bridge the two disciplines. We hypothesize that padel is less physically demanding than tennis due to its smaller playing space, the use of walls to sustain rallies, lower ball speed and racket design. Accordingly, this study aims to compare the performance demands and game characteristics of padel and tennis doubles during cross-over competition.

## 2 Material and methods

### 2.1 Subjects

Eight (*n* = 8) male (age of 27.0 ± 7.4 years, height of 186.3 ± 7.7 cm, body mass of 81.5 ± 10.7 kg, training frequency of tennis 4.8 ± 1.6 and padel 4.9 ± 1.4 h/week) participated in this study. On average, they had 20.5 ± 7.5 years of tennis experience and 3.4 ± 1.1 years in padel. Each participant was classified at the 6.0 level according to the National Tennis Rating Program (NTRP), which is standardized based on performance in major national singles and doubles tournaments. Additionally, all participants were ranked at the A-level in padel according to the Lithuanian national ranking system. Each provided written informed consent. The study protocol was approved by the local Institutional Research Ethics Committee (SA-EK-24-53) and followed the ethical guidelines outlined in the Declaration of Helsinki.

### 2.2 Experimental procedure design

A total of 12 simulated matches were conducted during the study period. These matches consisted of six doubles tennis (TEN) and six padel (PAD) sessions. The sessions were analysed to assess various performance metrics, including player movement, shot accuracy, and overall match strategy. Doubles teams were formed according to participants' skill levels in both TEN and PAD, ensuring a balanced competitive environment. All players competed in a round-robin format, facing a variety of opponents. Each participant played three TEN and three PAD matches, each lasting 40 min simulating one game set (23). The TEN matches were scheduled on 1 day, while the PAD matches occurred on another to ensure similar physical conditions across both formats. To maintain consistency in environmental factors, all matches were played simultaneously on two separate courts. After each match, participants had a 30-minute passive recovery period. The TEN and PAD matches were conducted on open-air fibrillated artificial grass courts with sand infill to ensure consistent playing conditions, particularly regarding player movement and ball bounce. The weather conditions were similar for both sets of matches, with an air temperature of 22°C, wind speeds of 2 m/s, and relative humidity of 68%. Prior to each match, participants completed a 15-minute warm-up, which included rallies and serves to prepare them for the upcoming match. The matches followed the official rules of the International Tennis Federation (ITF) and the International Padel Federation (FIP), including changeovers after every odd-numbered game. Players also officiated the matches, following self-refereeing protocols. Each match used a set of three new ITF- and FIP-approved balls. Players retrieved balls between points to maintain match flow and adhere to standard play protocols.

#### 2.2.1 Data collection and processing

##### 2.2.1.1 External load data collection

External load variables were quantified using VXSport inertial measurement units (VXSport, Wellington, New Zealand), which recorded data at a frequency of 100 Hz (24). These devices were securely positioned between the scapulae using the manufacturer's vest, ensuring optimal placement prior to the simulated TEN and PAD matches. The triaxial units, which incorporate gyroscopes, accelerometers, and magnetometers, captured movement across all three spatial axes. The devices provided comprehensive data on both the volume and intensity of the external load. Key metrics included Distance Rate (DR, in m·min^−^¹), Maximum Speed (MS in km·h^−^¹), Average Speed (AS in km·h^−^¹), Total Sprints (TS, as number) (>15.00 km·h^−^¹), High-Intensity Accelerations (ACC) and Decelerations (DEC, as number) (>3 m·s^−2^), and the distance covered during ACC and DEC (measured in meters). Total Distance Covered (TDC) was also recorded in meters. To evaluate varying movement intensities, speed zones were defined according to the locomotive categories established by Kilit and Arslan (14). Distances traveled within each of the four defined speed zones were categorized as follows: DSZ 1 (0.00–7.00 km·h^−^¹), DSZ 2 (7.01–12.00 km·h^−^¹), DSZ 3 (12.01–18.00 km·h^−^¹), and DSZ 4 (>18.01 km·h^−^¹).

VXSport devices were utilized to monitor continuous HR measurements using Suunto HR sensors (Suunto Smart Sensor, Suunto, Oy, Finland) (25). These sensors were integrated into a vest designed for strapless HR monitoring. The HR devices were positioned between the scapulae, alongside VXSport inertial measurement units, enabling precise and reliable HR data acquisition while ensuring athlete comfort. HR data were stored on VXSport devices via Bluetooth connectivity and analysed to determine HR metrics, including average HR (HRavg, bpm), maximum HR (HRmax, bpm), and resting HR (rest HR, bpm). Following data collection, external load and HR metrics were downloaded, stored, and processed using VXSport software (version 7.1.0.4). In addition to HR monitoring, capillary blood samples were collected from the player's fingertip 3 min after the end of TEN and PAD matches. These samples were immediately analysed for blood lactate concentration using a validated lactate analyser (Lactate Pro; Arkray, Tokyo, Japan). Blood lactate levels serve as key indicators of anaerobic effort, helping to assess physiological stress and recovery status.

##### 2.2.1.2 Simulated match variables collection

All TEN and PAD matches were recorded using two digital cameras (GoPro Hero9 Black). These cameras were positioned 2.5 m above the court and 5.5 m away from the tennis courts and 1.5 m above and 3 m away from padel courts, to provide an optimal and unobstructed view for analysis. The data collection process for simulated match variables followed established methodologies from prior research (26). The following key variables were systematically analysed using LongoMatch® software (LongoMatch® version 1.5.9, Barcelona, Spain): (a) total number of Rallies per Match (RPM); (b) Rally Duration (RD measured in seconds, from the server's initial strike to the conclusion of the point, as defined in the rules); (c) number of Total Strokes (TS); (d) number of Strokes per Rally (SPR); (e) 1st Serve points won %; (f) 2nd Serve points won %; (g) Points per Game (PPG); (h) Average Rest Interval between rallies (ARI, measured in seconds, this refers to the time interval from the end of one point to the start of the next). The analysis also included a detailed assessment of shot variety and execution, providing insight into player strategies and performance: (a) Serve efficiency (1st SE % and 2nd SE %); (b) Volleys: forehand (FV), backhand (BV), and smash (SV); (c) Groundstrokes (GS): forehand (F), backhand (B), and lobs (L). For padel matches, additional shot categorization was included for shots played off the wall. These were defined as instances when the ball, after bouncing on the ground, rebounded off the wall. Each of these shots was assigned to the appropriate variable for detailed analysis. To ensure the accuracy and consistency of the analysis, two researchers with expertise in tennis and padel coaching and performance analysis conducted the evaluations. The inter-rater reliability was confirmed to be high, with kappa coefficients exceeding 0.90, which reinforces the robustness and reliability of the data and methods employed.

### 2.3 Statistical analysis

A descriptive analysis was conducted to summarize the dataset, reporting means and standard deviations for all relevant variables. Subsequently, a linear mixed model (LMM) was employed to compare TEN and PAD while accounting for repeated measures within athletes. The model included match performance, shot variety, and execution as dependent variables, while sport type (TEN vs. PAD) was specified as a fixed effect. To control for inter-individual variability, player identity was included as a random effect, with a random intercept to account for baseline differences among players, while assuming a fixed slope for the effect of the sport. Statistical significance was assessed using an alpha level of 0.05 (*p* < 0.05), and all computations were performed using Jamovi software (version 1.2.27, 2020).

## 3 Results

A comparative analysis of match performance variables between PAD and TEN is presented in Table 1. LMM analysis revealed significant differences across all external load and physiological parameters. Tennis matches demonstrated substantially greater DR (TEN: 43.92 ± 3.93 m/min vs. PAD: 34.79 ± 6.60 m/min, *p* < 0.001), as well as higher AS (2.63 ± 0.25 km·h^−^¹) and MS (17.78 ± 3.07 km·h^−^¹), confirming the more intense locomotor demands of tennis. Similarly, the TS and ACC and DEC events were all significantly higher in TEN compared to PAD (e.g., ACC: 20.04 ± 8.40 vs. 13.58 ± 7.96; *p* = 0.006), as was TDC: 1,706.83 ± 181.92 vs. 1,392.96 ± 256.09 m; *p* < 0.001. Movement distribution across speed zones also favored tennis, with significant differences in each zone (DSZ1–DSZ4; all *p* < 0.001). With respect to physiological responses, tennis elicited significantly higher HRavg (129.13 ± 17.68 vs. 123.96 ± 17.43 bpm; *p* = 0.009) and HRmax (164.04 ± 12.86 vs. 155.33 ± 15.02 bpm; *p* < 0.001). However, no statistically significant difference was observed in post-match Bla concentrations between the two sports, although values were slightly elevated in TEN (5.38 ± 3.07 mmol·L^−^¹) compared to PAD (4.23 ± 2.41 mmol·L^−^¹). These results indicate that TEN is characterized by higher external workload and greater cardiorespiratory strain, despite comparable internal metabolic load indicators.

**Table 1:** Comparison of match performance between TEN and PAD players.

Variables	Padel	Tennis	AIC	R-squared conditional	Estimate (95% CI)	SE	*p*-value
DR (m/min)	34.79 ± 6.60	43.92 ± 3.93	297.169	0.614	9.13 (6.60, 11.70)	1.29	<0.001
AS (km·h^−^¹)	2.10 ± 0.40	2.63 ± 0.25	30.844	0.588	0.54 (0.38, 0.70)	0.08	<0.001
MS (km·h^−^¹)	15.23 ± 2.69	17.78 ± 3.07	243.960	0.166	2.55 (0.92, 4.18)	0.83	0.004
TS (num)	9.67 ± 5.21	15.63 ± 8.10	323.677	0.333	5.96 (2.49, 9.43)	1.77	0.002
ACC (num)	13.58 ± 7.96	20.04 ± 8.40	342.832	0.253	6.46 (2.13, 10.80)	2.21	0.006
DEC (num)	1.08 ± 1.28	1.92 ± 1.53	174.359	0.193	0.83 (0.08, 1.59)	0.38	0.036
ACC (m)	50.27 ± 31.06	76.27 ± 34.03	299.653	0.247	26.00 (3.99, 48.00)	11.23	0.029
DEC (m)	3.07 ± 3.62	5.27 ± 5.12	180.131	0.156	2.20 (−0.83, 5.23)	1.54	0.167
TDC (m)	1,392.96 ± 256.09	1,706.83 ± 181.92	658.355	0.507	314.00 (230.00, 425.00)	56.60	<0.001
DSZ1 (m)	1,004.63 ± 137.96	1,176.54 ± 93.75	599.911	0.393	172.00 (107.00, 237.00)	33.00	<0.001
DSZ2 (m)	362.46 ± 127.86	476.00 ± 150.25	605.405	0.499	114.00 (52.10, 175.00)	31.30	<0.001
DSZ3 (m)	24.13 ± 14.21	45.58 ± 25.20	429.424	0.371	21.50 (11.00, 31.90)	5.34	<0.001
DSZ4 (m)	0.92 ± 2.30	7.58 ± 8.28	313.044	0.378	6.67 (3.55, 9.79)	1.59	<0.001
HRavg (bpm)	123.96 ± 17.43	129.13 ± 17.68	352.340	0.874	5.17 (1.46, 8.87)	1.89	0.009
HRmax (bpm)	155.33 ± 15.02	164.04 ± 12.86	351.600	0.795	8.71 (4.83, 12.60)	1.98	<0.001
Bla (mmol·L^−1^)	4.23 ± 2.41	5.38 ± 3.07	234.914	0.301	1.16 (−0.19, 2.51)	0.69	0.100

Notes: *t*, difference between sample means; *p*, between group-subject effect; DR, distance rate; m/min, meters per minute; AS, average speed; km·h^−^¹, per hour; SM, speed maximum; TS, sprints total; num, number; ACC, high intensity acceleration (>3 m·s^−2^); DEC, high intensity deceleration (>3 m·s^−2^); ACC D, high intensity acceleration distance; m, meters; DEC D, high intensity deceleration distance; TDC, total distance covered; DSZ 1, distance speed zone (0.00–7.00 km·h^−^¹); DSZ 2, distance speed zone (7.01–12.00 km·h^−^¹); DSZ 3, distance speed zone (12.01–18.00 km·h^−^¹); DSZ 4, distance speed zone (>18.01 km·h^−^¹); HRavg, heart rate average; bpm, beats per minute; HRmax, heart rate maximum; Bla, blood lactate; mmol·L^−1^, millimoles per liter.

Table 2 provides a comparison of technical-tactical performance between the two sports. PAD players demonstrated higher serve efficiency, with significantly greater first (80.17 ± 9.30%) and second (90.96 ± 13.41%) serve success rates compared to TEN players (first: 61.17 ± 9.54%, second: 75.54 ± 18.56%; both *p* < 0.01). PAD also featured a higher frequency of net actions, including FV, BV, and SV, with all measures significantly greater in PAD (e.g., SV: 9.71 ± 3.16 vs. 0.63 ± 0.82; *p* < 0.001). In terms of groundstrokes, padel players executed more forehands, backhands, and notably L, with a large disparity in lobs (20.50 ± 8.04 vs. 2.83 ± 2.62; *p* < 0.001), reflecting the tactical structure of PAD play. Additionally, PAD matches featured a significantly higher number of RPM (92.33 ± 17.62 vs. 67.17 ± 2.16; *p* < 0.001), as well as longer RD (6.50 ± 0.33 s) and more SPR (4.53 ± 0.28), indicating a greater volume of continuous play. ARI were significantly shorter in PAD (17.36 ± 2.72 s) than in TEN (29.67 ± 1.78 s; *p* < 0.001), reinforcing the notion of PAD as a more continuous, rhythmically demanding sport. These findings emphasize that PAD imposes a higher technical workload and requires sustained tactical engagement, whereas TEN involves greater intensity in movement and recovery intervals, reflecting different performance profiles despite overlapping physiological responses.

**Table 2:** Comparison of stroke variety and execution performance between TEN and PAD players.

Variables		Padel	Tennis	AIC	R-squared conditional	Estimate (95% CI)	SE	*p*-value
Serve	1st SV %	80.17 ± 9.30	61.17 ± 9.54	350.141	0.524	−18.90 (−24.3, −13.6)	2.71	<0.001
	2nd SV %	90.96 ± 13.41	75.54 ± 18.56	409.754	0.195	−15.40 (−24.5, −6.29)	4.66	0.002
Volleys (num)	FV	14.17 ± 6.03	5.21 ± 2.78	290.701	0.486	−8.96 (−11.05, −6.31)	1.35	<0.001
	BV	8.54 ± 3.50	5.04 ± 2.27	245.986	0.302	−3.50 (−5.13, −1.87)	0.83	<0.001
	SV	9.71 ± 3.16	0.63 ± 0.82	222.379	0.798	−9.08 (−10.39, −7.78)	0.67	<0.001
GS (num)	F	26.92 ± 7.86	22.75 ± 8.83	337.673	0.402	−4.17 (−8.00, −0.34)	1.95	0.039
	B	21.79 ± 7.72	13.38 ± 4.81	319.474	0.4903	−8.42 (−11.8, −5.03)	1.73	<0.001
	L	20.50 ± 8.04	2.83 ± 2.62	313.874	0.690	−17.7 (−21.05, −14.3)	1.73	<0.001
RPM (num)		92.33 ± 17.62	67.17 ± 2.16	385.052	0.506	−25.2 (−32.3, −18.1)	3.62	<0.001
RD (s)		6.50 ± 0.33	4.87 ± 0.51	61.437	0.801	−1.63 (−1.86, −1.39)	0.12	<0.001
TS (num)		81.17 ± 18.20	46.42 ± 7.51	394.996	0.619	−34.8 (−42.6, −26.9)	4.00	<0.001
SPR (num)		4.53 ± 0.28	3.60 ± 0.54	61.002	0.598	−0.94 (−1.17, −0.71)	0.12	<0.001
1st Serve points won %		59.96 ± 10.87	64.00 ± 15.13	389.189	0.115	4.04 (−3.64, 11.20)	3.64	0.273
2nd Serve points won %		61.13 ± 9.61	46.83 ± 14.95	379.458	0.470	−14.3 (−20.3, −8.26)	3.08	<0.001
PPG		5.66 ± 0.28	5.75 ± 0.14	−2.933	0.05	0.09 (−0.03, 0.22)	0.06	0.157
ARI (s)		17.36 ± 2.72	29.67 ± 1.78	222.063	0.880	12.30 (11.00, 13.60)	0.66	<0.001

Notes: *t*, difference between sample means; *p*, between group-subject effect; 1st SV %, first serve efficiency; 2nd SV %, second serve efficiency; FV, forehand volley; BV, backhand volley; SV, smash; GS, groundstrokes; *n*, number; F, forehand; B, backhand; L, lob; RPM, Rallis per match; RD, Rallies duration; s, seconds; TS, total strokes; SPR, strokes per rally; PPG, points per game; ARI, average rest interval.

## 4 Discussion

This study investigated the performance demands and game characteristics of PAD and TEN during cross-over competition. The findings demonstrated that TEN imposes greater physical demands than PAD. This conclusion is supported by metrics such as distance rate, average speed, number of sprints, accelerations, decelerations, total distance covered, and distances achieved within specific speed zones. Physiologically, TEN elicited more pronounced responses, including higher maximal cardiorespiratory demand and increased reliance on glycolytic energy systems. From a technical perspective, the game characteristics of PAD and TEN exhibited distinct patterns. PAD players showed greater serve efficiency, performed more volleys, backhands, and lobs, and engaged in longer rallies compared to their TEN counterparts. In contrast, TEN games featured extended average rest intervals, reflecting differences in pacing strategies and recovery demands.

### 4.1 Performance demands

**Figure 1:**
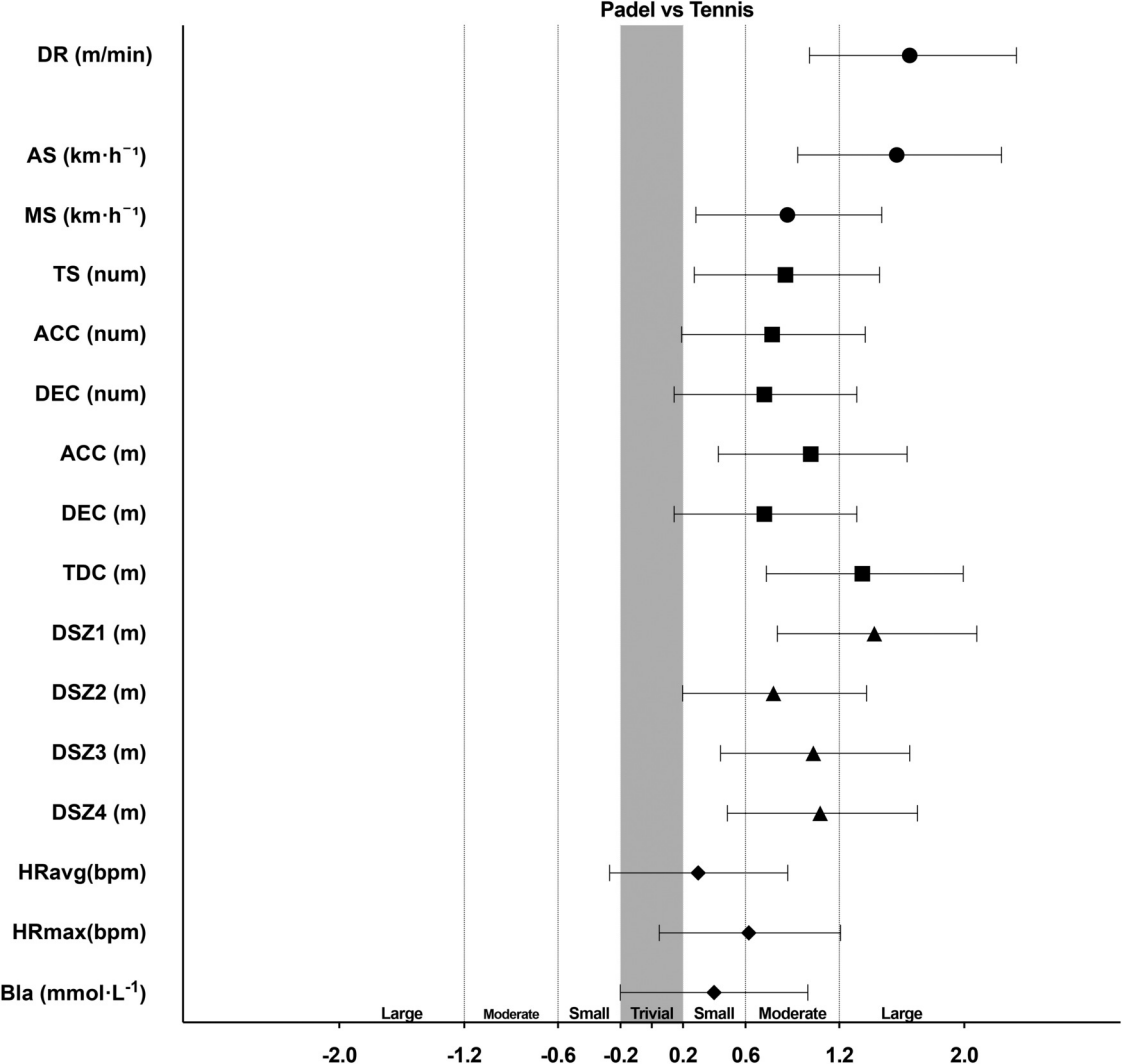
Standardized (Cohen) differences in performance demand variables during games. Error bars indicate uncertainty in the true mean changes with 95% confidence intervals. Abbreviations: DR, distance rate; MS, maximum speed; TS, total sprints; ACC, acceleration, DEC, deceleration; TDC, total distance covered; DSZ, distance speed zone; HRavg, heart rate average; HRmax, heart rate maximum; Bla, blood lactate.

Previous research on racket sports has primarily used systematic reviews to examine match load characteristics, often relying on notational data (3, 5). Our simulated match study revealed that the distance rate in TEN was higher than in PAD (Table 1, and Figure 1). This difference can be attributed to several factors, including the larger court dimensions in tennis, the additional space behind the baseline, and the greater speed of the ball. Studies have consistently emphasized the significant impact of court size on players' external and internal loads (27, 28). Another critical distinction lies in the ball's flight and rebound mechanics. These dynamics are unique in padel, influenced by the equipment and the integration of walls as part of gameplay (29). Our findings also showed that AS and MS were significantly higher in TEN compared to PAD. Specifically, the AS in TEN was 2.63 ± 0.25 km·h^−^¹, aligning with the reported average speed of adult singles tennis players on clay courts (2.2 ± 0.3 km·h^−^¹) (30). In contrast, Castillo-Rodríguez et al. (3) found that Spanish lower-level PAD players recorded higher AS values than their middle- and higher-level counterparts during matches, with speeds of 1.93 ± 0.31, 2.13 ± 0.25, and 2.18 ± 0.31 km·h^−^¹, respectively. Interestingly, the AS of our PAD players (2.11 ± 0.39 km·h^−^¹) closely matched that of Spanish middle-level players, highlighting the influence of skill level on match dynamics.

Our study is the first to analyze the TS count when player speed exceeds 15.00 km·h^−^¹, providing new insights into high-intensity demands in racket sports. In TEN, TS was higher compared to PAD, confirming the elevated intensity levels in tennis matches. While PAD players often exert substantial effort, their ability to reach maximal speed is constrained by the smaller court dimensions and space limitations, factors akin to those observed in small-sided training games (27). This spatial limitation is critical when comparing the overall intensity and performance characteristics of both sports. Both TEN and PAD are characterized by periods of intense work, followed by relatively long rest intervals (31). Thus, ACC and DEC are key variables for assessing intensity, as they offer insights into the timing and nature of player exertion. In elite team sports, high-intensity acceleration and deceleration during match play typically range from 0.5 to over 3 m·s^−^² (32). In our study, we used a range of velocity indicators, defining ACC and DEC events when acceleration or deceleration exceeded 3 m·s^−^². The number of ACC events was higher in TEN than in PAD (*p* = 0.009), with tennis players covering greater distances during ACC (*p* < 0.001). Hoppe et al. (31) found that most of the time and distance covered during ACC and DEC in tennis occurs within the range of −1 to 1 m·s^−^², which accounts for 93.7% of the time and 89.6% of the distance. Therefore, our study focused on the top 10% of high-intensity data, providing critical yet under-explored insights into the demands of the sport. Previous studies have also shown that ACC distance in tennis typically exceeds DEC distance within the −1 to 1 m·s^−^² range (30, 31). In contrast, due to the >3 m·s^−^² velocity threshold applied in our study, we found that the ACC values—both in terms of event count and distance—were substantially greater than those for DEC.

Our analysis revealed that during equal playing time, the TDC in TEN exceeded that in PAD by over 300 m (*p* = 0.0001), underscoring a significant difference in external load between the two sports. This discrepancy may, in part, be attributed to the additional distance tennis players cover while retrieving balls, a factor not as prevalent in padel. Castillo-Rodríguez et al. (3) observed that high-level PAD players covered less distance than their middle- and lower-level counterparts during set and match play. In their simulation, PAD players covered 1,402.21 ± 256.11 m, whereas tennis players covered 1,706.54 ± 181.93 m. Previous studies indicate that a PAD match typically covers approximately 1,813.7 ± 745.7 m (3), while a tennis singles match can range from 1,989 ± 346 to 3,569 ± 532 m (33). In both sports, TDC was distributed across four distinct speed zones, with tennis players covering significantly more distance in each zone compared to PAD players. Approximately 70% of the distance in both sports was covered in DSZ1, around 25% in DSZ2, approximately 2% in DSZ3, and roughly 0.5% in DSZ4.

The mean HRavg did not show a statistically significant difference between PAD and TEN, suggesting similar cardiorespiratory demands during simulated match play. Mas et al. (34) reported a HRavg of 153.7 ± 14.6 bpm in international-level PAD players, which is notably higher than the HRavg observed in our study. This discrepancy may reflect differences in player conditioning, match context, or methodological approaches. In contrast, García et al. (35) found an HRavg of 126.8 ± 10.4 bpm in recreational-level PAD players, which aligns closely with our result of 123.79 ± 17.58 bpm. This consistency across studies suggests that the cardiorespiratory demands of simulated match play in PAD are comparable across skill levels. Kilit et al. (33) reviewed HRavg performance demands in TEN singles, reporting values ranging from 141 ± 19 to 164 ± 15 bpm, which are considerably higher than the 129.13 ± 17.68 bpm observed in our study. This difference may be attributed to the varying intensities and match characteristics in real match play compared to our simulated conditions. The observed difference in HR between simulated and real match conditions can likely be attributed to variations in psychophysiological demands, particularly those associated with autonomic nervous system activation (36). Furthermore, the higher HRmax observed in tennis compared to PAD may be linked to external load factors, such as greater movement variability, higher sprint intensities, and more frequent high-impact actions, which are typically more pronounced in TEN due to its specific performance demands and match-play characteristics.

BLa did not differ between conditions, with both exceeding the anaerobic threshold. This suggests that glycolytic energy production was comparably intense in both scenarios. Previous studies investigating PAD players across varying skill levels reported an average BLa of 2.87 ± 1.48 mmol, which was lower than the values observed in our study (3). A similar study found no significant difference in BLa between PAD and TEN, although their values were also lower than those documented in our research (19). The considerable variability in BLa suggests that lactate metabolism is highly individualized, underscoring the need for more in-depth research to better understand these discrepancies.

### 4.2 Game attributes

**Figure 2:**
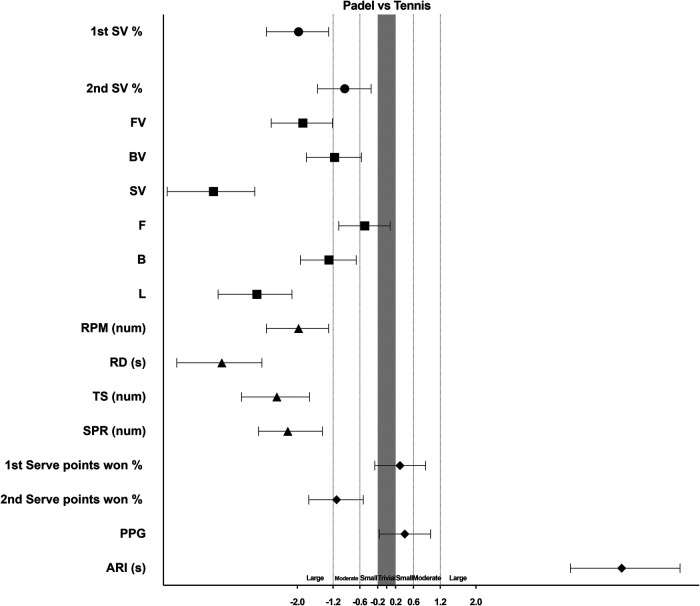
Standardized (Cohen) differences in game attributes. Error bars indicate uncertainty in the true mean changes with 95% confidence intervals. Abbreviations: 1st SV %, first serve efficiency; 2nd SV %, second serve efficiency; FV, forehand volley; BV, backhand volley; SV, smash; F, forehand; B, backhand; L, lob; RPM, rallis per match; n, number; RD, rallies duration; s, seconds; TS, total strokes; SPR, strokes per rally; PPG, points per game; ARI, average rest interval.

Volleys and SV are foundational to doubles play, influencing both strategy and point outcomes. PAD players consistently demonstrate superior performance in FV, BV, SV compared to their TEN counterparts (Table 2, and Figure 2). In padel, players typically advance toward the net following a serve, capitalizing on the tactical advantage this position offers to increase their chances of success. Research highlights PAD emphasis on fast-paced net play and the frequent use of serves to control the tempo of the game (37). In tennis doubles, volleys are similarly strategic but are executed less frequently than in padel. This difference may stem from several factors, including opponents' strategies, court surfaces, and individual playing styles (38). The distinct tactical demands of each sport require tailored GS strategies. In padel, players often transition from defence to offense, emphasizing precision and shot placement. The smaller court dimensions and presence of walls in padel encourage the use of F, B, L, and drop shots to maintain control (26). In contrast, in tennis, L are primarily defensive or counterattacking, while GS emphasize baseline dominance, with powerful, spin-heavy F dictating rallies (39). These differences underscore the need for sport-specific training, with PAD players focusing on advanced net play strategies, while TEN doubles players should develop a balanced approach, mastering both baseline dominance and adaptable net play tactics.

A key distinction between PAD and TEN lies in RD and SPR. In PAD, rallies tend to be longer due to the smaller, enclosed court and the use of walls, which facilitate shot recovery. Defensive strategies such as wall bounces and lobs are frequently employed by players to extend rallies and gain additional time (26). In contrast, TEN rallies are generally shorter because of the larger court dimensions, which, when combined with aggressive baseline play, power, and spin, often lead to quicker point conclusions (3). The current study reports average RD (4.87 ± 0.51 s) and SPR (3.60 ± 0.54) for TEN, which aligns with previous research showing RD (3.45 ± 2.91 s) and SPR (3.40 ± 2.30) (18). However, in PAD, RD (6.50 ± 0.33 s) and SPR (4.53 ± 0.28) differ from typical match characteristics, where RD is reported as (8.90 ± 0.61 s) and SPR at (6.10 ± 0.50 shots) (40). These discrepancies are likely influenced by factors such as game level, playing style, and match conditions. These findings underscore the unique physiological demands of each sport. PAD demands greater endurance and the ability to sustain high-intensity exchanges, while TEN places a premium on explosive power (41). The fast-paced nature of PAD enables quicker transitions between rallies, resulting in shorter ARI (40). In contrast, TEN, with its larger court and longer transitions (such as walking between points), typically involves longer ARIs (42).

Nevertheless, some limitations may be acknowledged. One notable limitation of this study is the participants' relatively limited experience in padel compared to tennis, which may have impacted their technical execution and tactical decision-making during gameplay.

## 5 Conclusion

This study provides valuable insights into the distinct physical and tactical demands of PAD and TEN, revealing key differences in movement patterns, rally characteristics, and physiological responses. TEN is marked by higher movement speeds, greater sprint frequency, and extended recovery periods, emphasizing the need for explosive power and anaerobic conditioning. In contrast, PAD is characterized by prolonged rallies, frequent volleys, and sustained play, highlighting the importance of endurance and tactical adaptability. Despite these contrasting gameplay dynamics, both sports impose similar physiological demands, as reflected in comparable heart rate responses and metabolic reliance. These findings underscore the necessity of sport-specific training programs, with TEN players benefiting from sprint and recovery-focused conditioning, while PAD athletes should prioritize endurance and sustained technical execution. Future research should explore longitudinal adaptations to training in each sport, further optimizing performance strategies for athletes and coaches.

## Data Availability

The raw data supporting the conclusions of this article will be made available by the authors, without undue reservation.
