# Correction: Padel vs. tennis doubles: a comparison of performance demands and game attributes

**DOI:** 10.3389/fspor.2025.1663379

**Published:** 2025-08-29

**Authors:** Rūtenis Paulauskas, Domantas Šakinis, Bruno Figueira

**Affiliations:** ^1^ Educational Research Institute, Education Academy, Vytautas Magnus University, Kaunas, Lithuania; ^2^ Departamento de Desporto e Saúde, Escola de Saúde e Desenvolvimento Humano, Universidade de Évora, Évora, Portugal; ^3^ Comprehensive Health Research Centre (CHRC), Universidade de Évora, Évora, Portugal

**Keywords:** external load, speed zones, groundstrokes, volleys, rallies

In the published article, an incorrect symbol was mistakenly used in the abstract.

**The original sentence read:**

“All variables compared between Tennis and Padel showed statistical differences (*p* > 0.05)”

**This has been corrected to read:**

“All variables compared between Tennis and Padel showed statistical differences (*p* < 0.05)”

The original version of this article has been updated.

